# Promoting Resilience in Healthcare Workers: A Preventative Mental Health Education Program

**DOI:** 10.3390/ijerph21101365

**Published:** 2024-10-16

**Authors:** Scarlett S. Ho, Wuraola Sosina, Jonathan M. DePierro, Stefanie Perez, Aysha Khan, Sydney Starkweather, Deborah B. Marin, Vansh Sharma, Jonathan A. Ripp, Lauren A. Peccoralo, Dennis S. Charney

**Affiliations:** 1 Department of Psychiatry, Icahn School of Medicine at Mount Sinai, New York, NY 10029, USA; wuraola.sosina@mssm.edu (W.S.); jonathan.depierro@mssm.edu (J.M.D.); stefanie.perez@mountsinai.org (S.P.); aysha.khan@mssm.edu (A.K.); starkweathersydney@gmail.com (S.S.); deborah.marin@mssm.edu (D.B.M.); vansh.sharma@mssm.edu (V.S.); dennis.charney@mssm.edu (D.S.C.); 2 Department of Psychiatry and Behavioral Sciences, Feinberg School of Medicine, Northwestern University, Chicago, IL 60208, USA; 3 Office of Well-Being and Resilience, Icahn School of Medicine at Mount Sinai, New York, NY 10029, USA; jonathan.ripp@mssm.edu (J.A.R.); lauren.peccoralo@mssm.edu (L.A.P.); 4 Departments of Neuroscience, Icahn School of Medicine at Mount Sinai, New York, NY 10029, USA; 5 Department of Pharmacological Sciences, Icahn School of Medicine at Mount Sinai, New York, NY 10029, USA

**Keywords:** healthcare workers, mental health, resilience-building, educational workshops, coping skills

## Abstract

Healthcare workers face greater risks for mental health conditions and chronic stress due to the demanding nature of their roles. The COVID-19 pandemic exacerbated these challenges and increased vulnerabilities to long-term mental health conditions. The present study adapts an existing resilience-based educational workshop program to address the time constraints and unique needs of the healthcare workforce in a post-COVID-19 world. Expanded from its initial focus on resilience, the workshop curriculum incorporates psychoeducation on common mental health issues. Between July 2022 and June 2024, a 15 min “huddle” format offered on-site training to equip HCWs in a large urban health system with coping strategies to prevent, manage, and recover from stress. Attendance and anonymous feedback were collected at each session via brief electronic surveys. Participant responses (*n* = 1403) obtained immediately post-huddle suggested positive impact on stress management, perceived leadership support, and resilience. Findings show the potential of brief huddles to improve mental health and resilience in healthcare workers and similar workforces. Our findings support the efficacy of brief, evidence-based educational huddles in enhancing resilience, mental health awareness, and coping skills among HCWs. This model holds significant potential for widespread implementation across healthcare and other high-stress workplaces.

## 1. Introduction

Healthcare workers (HCWs) are at heightened risk for mental health conditions, chronic stress, and burnout compared to other occupations [1]. The demanding nature of their work, characterized by long hours, heavy patient loads, and exposure to life-threatening situations, creates a high-stress environment. Frequent exposure to emergencies, disasters, and violence further compounds emotional strain [2]. Beyond their professional lives, HCWs juggle personal and family responsibilities, such as childcare, financial strain, and relationships [3]. The COVID-19 pandemic amplified these stressors, deteriorating the quality of both personal and professional protective factors [4], subsequently increasing vulnerability to depression, anxiety, posttraumatic stress disorder (PTSD), and other mental health disorders [5,6].

The COVID-19 pandemic had a profound impact on the healthcare workforce. A 2022 National Nursing Workforce Survey of nearly 30,000 nurses found that 15.2% to 44.8% of nursing staff considered leaving the profession due to fatigue, emotional exhaustion, and burnout [7]. The pandemic’s impact, coupled with ongoing staffing shortages, highlights the urgent need for healthcare institutions to address these systemic issues. The American Medical Association has called upon healthcare organizations to adopt a systems-based approach to becoming resilient organizations [8], urging them to take action to address acute and chronic stress faced by HCWs. Recommendations included reducing psychological trauma (before, during, and after a crisis), preventative measures to support well-being, and treatment to address mental health conditions of HCWs [9].

In response to the needs made apparent by the pandemic, the Center for Stress Resilience and Personal Growth (CSRPG) was launched in June of 2020 as a dedicated resource for Mount Sinai Health System (MSHS) staff, faculty, students, and trainees [10]. CSRPG provides educational resources, cognitive behavioral therapy, and if indicated, pharmacotherapy [11] available to the entire workforce. In the summer of 2020, CSRPG developed a series of resilience-promoting educational workshops aimed to equip HCWs with healthy coping strategies for preventing, managing, and recovering from stress and crises. Workshop content aligns with evidence-based resilience factors identified by Southwick and Charney [12], including realistic optimism, facing fears, active coping, and self-care [13]. Initially, resilience-promoting educational workshops were offered to frontline HCWs during breaks or post-shift hours. Each 60–90 min workshop was facilitated by CSRPG staff (psychiatrist, psychologist, or chaplain) and trained hospital leadership volunteers. Participants learned about a specific resilience factor, reflected on current challenges, and developed action plans to apply coping strategies they learned. Over time, workshop content evolved to address emerging workforce needs post-pandemic, such as managing moral distress. However, over time, it became clear that particularly vulnerable groups, including nurses, who as an occupation have been shown to experience high levels of burnout and mental health distress [14,15], may not have the ability to participate in such a program due to constraints on their time, work location, and patient care responsibilities.

To address both the need for timely education and the practical constraints of many health system staff, we developed a resilience-focused program in the form of a brief “huddle” format meant to be deployed in vivo on medical units. In this paper, we describe the adaptation, implementation, and self-reported outcome data from this program deployed over two years.

## 2. Materials and Methods

The current study is part of a larger federally funded HRSA [Health Resources and Services Administration] CARE (Culture, Access, Resilience, and Education) grant aimed at decreasing burnout, promoting resiliency, and mitigating emotional distress in the healthcare workforce. CARE was designed to address burnout and mental health challenges in the healthcare workforce, encompassing leadership skills enhancement, resilience and mental health training, mental health screening and treatment, and system-level wellbeing-focused communications. Only the resilience and mental health training arm will be described in the current paper.

To enhance accessibility and expand the program reach, the original 60–90 min workshop format was condensed into 15 min on-site “huddles” for frontline HCWs. A similar “briefing” format was implemented for hospital security personnel and supervisors [16]. By reducing time commitments and offering training at the point of care, these formats addressed common barriers to participation and increased ease of access for more HCWs to participate. Huddles were conducted by the same facilitators to ensure consistency, maintain continuity in discussions, and foster trust among huddle participants. The huddle curriculum evolved from its initial focus on resilience factors to encompass a wider range of evidence-based interventions. These included psychological first aid, managing moral distress, psychoeducation modules on common mental health challenges faced by HCWs (e.g., depression and suicide risk, anxiety, and substance use), and a mid-point review huddle. See Table 1 for huddle topics.

High-risk areas within the hospital system, including emergency departments and intensive care units, were identified based on previous well-being, mental health, and resilience survey data [14,17]. CSRPG psychologists and social workers collaborated with area leadership to assess specific needs and introduce the huddle program. With leadership support, CSRPG facilitators conducted weekly, 15 min huddles across morning, afternoon, and evening shifts in targeted areas. Over 13 weeks, 12 topics (e.g., Trust and Team Building, Facing Fears with Active Coping, Addressing Social Justice, Recognizing Depression and Risk for Suicide) and a mid-course review were delivered. Participants were encouraged to incorporate group well-being and resilience-building exercises into daily team meetings. Facilitators also provided information to participants about accessing additional mental health support, including individual screening and confidential mental health treatment.

### 2.1. Measures

Participants were asked to complete two brief surveys, an attendance form at the beginning and a separate anonymous feedback form at the end of the huddle, using a QR code linked to a Research Electronic Data Capture (Redcap) survey platform. The attendance form prompted participants to provide demographic information (e.g., role, discipline, unit, gender, etc.). The post-huddle feedback form, which was anonymous, asked participants five questions about their experience in the resilience huddle (e.g., the huddle gave me the tools to do my job well, the huddle gave me tools that are relevant to my life outside of work) rated on a Likert-type scale ranging from 0 (strongly disagree) to 4 (strongly agree). This set of feedback questions was adapted from prior work that aimed to engage hospital workers in a short period of time (approximately 1 min), reduce survey fatigue, and provide insight on participant satisfaction [18]. Free-text responses were also encouraged at the end of the feedback survey. The study was deemed exempt by the local Institutional Review Board, and participants were given a digital information sheet before participating.

### 2.2. Data Analysis

Data analysis employed a descriptive approach to examine participant demographics and huddle feedback. In the huddle feedback form, five Likert-type items ranging from 0 (strongly disagree), 1 (disagree), 2 (neutral), 3 (agree), and 4 (strongly agree) were used to assess participants’ experiences with the huddles. Following Sullivan and Artin’s [19] recommendations for analyzing and interpreting descriptive data, response frequencies were categorized as positive (strongly agree and agree), negative (strongly disagree and disagree), or neutral, to provide insights into the program’s impact.

## 3. Results

From July 2022 to June 2024, 332 huddles were completed with 1640 unique participants across the health system. Participants largely identified as female (78%), and nursing was the largest professional group (40%) represented in the data. See Table 2 for demographic information.

Of the 3307 times that participants attended the huddles, there was 50% (N = 1639) engagement in the anonymous feedback survey. For our analyses, we included only respondents who completed all the items in the survey (n = 1403; overall response rate = 42%). Through this survey, participants reported that these huddles provided them the tools to do their job effectively (81% agree/strongly agree) and were relevant to their life outside of work (80% agree/strongly agree). Participants also felt that the resilience huddles demonstrated a way in which hospital leadership valued them (72% agree/strongly agree), felt more confident in their ability to “bounce back” from life’s challenges after the resilience huddle (71% agree/strongly agree), and experienced reduced stress following the huddle (64% agree/strongly agree). See Table 3 for huddle ratings.

To contextualize these responses, we also examined the open-ended responses provided on the survey; of note, we did not utilize a formal qualitative analytic approach. The open-ended response section indicated that participants felt the huddles were useful in team building, increased individual and collective knowledge on coping, and provided new perspectives on approaching challenges. One participant described the huddle to be “very informative”, another expressed that the coping tools they learned helped them “prevent burnout”. Participants also expressed appreciation for the huddle facilitators, calling them “excellent”, “amazingly kind”, and noting that they “helped them a lot” (note that the examples provided in the huddles were relevant to their work and lives). Participants also valued the opportunity to share their challenging experiences with each other during the huddles; one participant stated they “really appreciate… the platform to have the safe space to talk to our friends and colleagues”, and other participants expressed that “it was refreshing to feel heard.” Some participants expressed different preferences for the timing of the huddles (beginning vs. end of shift) and discussed frustrations with chronic stressors (e.g., requesting “systemic changes” to address compensation and workload).

## 4. Discussion

As part of a broader workforce well-being initiative, we implemented a unit-based psycho-educational program for healthcare workers focused on mental health and resilience. The program’s goal was to provide timely resources to workers in high-stress settings who may not have been able to participate in supportive programming. Conducting the huddles in the work area for healthcare workers offered the advantages of convenience of location and minimization of disruption of their work. Additionally, this setting allowed facilitators to present educational materials that directly addressed the current challenges healthcare workers face, thereby improving skill acquisition and application among participants. Healthcare workers who participated in the resilience-promoting program reported increased knowledge of coping skills, which provided them with the tools to manage both professional and personal challenges, enhance resilience (e.g., have greater ability to recover from life’s difficulties), reduce stress, and increase sense of support. Research underscores the need for resilience programs to be adapted to the local context and schedules [20]. Our program emphasized brevity to address the time constraints faced by healthcare workers in a large urban healthcare system and offered content that is especially relevant to healthcare workers’ everyday challenges. Healthcare workers are prone to psychological distress due to the chronicity of work responsibilities, and this distress strongly predicts maladaptive coping strategies. Consequently, healthcare workers are more likely to engage in cognitive distortions, neglect hygiene and self-care practices, and use substances, which such coping strategies are associated with poorer mental health outcomes [21,22]. Our program focused on teaching adaptive coping strategies [23], including active coping, positive reframing, and understanding the breadth of social and emotional support. This aided to facilitate problem-solving, emotional management in high stress situations, and cognitive flexibility to promote subjective sense of control [24,25]. The brief resilience huddles thus provided HCWs with opportunities to expand their coping repertoire and adopt healthier mechanisms for managing challenges in positive and constructive ways.

Our outcome data also aligns with the growing literature on the role of workplace culture and leadership on employee well-being, over and above individual-level mental health interventions. Previous research suggests that HCWs in supportive environments experienced fewer mental health problems [26,27] and that feeling valued and supported lowers HCW’s risk of depression, anxiety, and PTSD [28]. Workers who feel respected and appreciated reported better health, job satisfaction, and lower turnover intentions [29,30]. Our data suggest that a brief, targeted intervention may show benefit in fostering a sense of community and leadership support, in the context of ongoing stressors and shifting healthcare landscape in the post-COVID era and offers avenues to improve occupational health across varying settings and disciplines. The program’s curriculum, community engagement strategies, and evaluation method offer a valuable framework adaptable to other healthcare settings or similar high-stress environments.

The format of program implementation and content provided a unique avenue for addressing mental health stigma and promoting literacy within a population where mental health stigma presents as a prominent barrier to care and recovery [31,32,33]. The literature demonstrates that successful interventions for addressing mental health stigma among healthcare professionals focus on social contact skills-based strategies and education, contextually relevant content, an opportunity to explore diverse perspectives, sufficient time, and organizational support [34]. The current program offered huddles in an in-person setting, allowing HCWs to connect with the huddle facilitators and their colleagues on their shared and unique experiences. Additionally, huddle content was relevant to the everyday experiences of HCWs, allowing them to directly apply the presented material and psychological resources to their roles and other areas of their lives. Importantly, our team worked collaboratively with leadership to ensure understanding and support for the program and the shared goal of promoting mental health and resilience in staff and throughout the health system. Often, employers and leadership find it difficult to gauge the scope of the mental health needs of their employees and report limited insight on best practices to provide employees with mental health resources and support [35,36,37]. Engaging leadership personnel to enhance their awareness of mental health needs and evidenced-based coping skills and best practices through such programs provides a framework for socially responsible care and encourages organizations foster a culture of mental health and well-being [38]. Champions of organizational mental health wellness have recommended that when implementing such programs, aims should emphasize supportive work culture, well-rounded mental health benefits, adequate mental health resources, structured workplace and practices, a healthy workplace environment, leadership support, utilization of outcomes measurement, and critical innovation [35]. The program described here is one offering of well-coordinated offices at our health system that aim to address all these recommendations.

Huddles provided participants with benefits that extended beyond mental health awareness and coping strategies. Participants indicated in their open-ended responses that these huddles allowed them to gain validation and support from their colleagues. Research indicates that social support in the workplace can mitigate the effects of negative factors within the work environment [39,40]. Specifically, support from colleagues enhances feelings of autonomy, competence, and relatedness, which in turn contribute to improved well-being, motivation, and job satisfaction [41,42,43]. Given the demanding schedules of healthcare workers, opportunities for breaks and reflection on workplace challenges are often limited. The huddles provided both formal and informal spaces that act as respites from work and promote employee connections, potentially mitigating burnout, reducing work-related distress, enhancing feelings of cooperation, and facilitating adaptation in uncertain circumstances [44,45]. Future research should explicitly investigate factors such as social support and job satisfaction as outcome measures.

### Limitations

There were several limitations to the present study. As an educational program, this study did not assess the mental health symptoms and wellbeing indicators pre- and post- huddles, which limits our knowledge of the quantitative psychological impact of this program. Further, we did not assess the longitudinal impact of these huddles on burnout or well-being with follow-up assessment points, limiting our knowledge to the immediate impact of the intervention. Future research should explore whether HCWs applied the content of the huddles to cope with their challenges, the long-term effectiveness of the coping skills taught in the program, and whether they decreased mental health stigma and increased utilization of mental health service in the context of a perceived need. Future research should incorporate qualitative studies, as they can offer unique insights into the impact of these huddles that qualitative methods may not capture.

## 5. Conclusions

While this study’s educational program offers valuable mental health support for frontline HCWs, a multifaceted approach is necessary for the long-term well-being of HCWs. Systemic issues causing acute and chronic stress (e.g., long hours, patient and staff safety concerns) must be addressed alongside resilience training and mental health education. Program evaluation of the current study is hindered by a lower-than-expected feedback survey completion rate, likely due to HCWs’ time limitations in clinical settings and potential survey fatigue. Future studies should examine the sustained effects of huddle participation on participants’ mental health knowledge and coping skill application over time to understand the long-term benefits of these huddles. Assessing the impact of resilience and mental health training on work culture, teamwork, and staff retention would also be of value. Lastly, with respect to replication and extension of this work, a train-the-trainer” model could ensure expertise transfer and adaptation, wherein unit leaders are empowered to lead huddles. Given the promising nature of such programs, healthcare organizations should prioritize allocating funding and resources for training.

Overall, our program provides a potential framework for providing preventative mental health and self-management content to an at-risk and vulnerable population who often underutilize mental health treatment services [31,32,33]. This study demonstrates scalable and effective avenues for delivering preventive interventions and addressing resource access disparities among diverse healthcare workers across various disciplines, genders, races, ages, and work locations. We recommend that healthcare organizations implement a comprehensive approach to mitigate the elevated mental health risks among their workforce by investing in preventive interventions that reduce stigma and expand access to mental healthcare services.

## Figures and Tables

**Table 1:** Huddle topics.

Resilience Coping Skills for Teams and Individuals	Psychoeducational Topics for Mental Health Awareness
Team Building and Trust	Depression and Suicide Awareness
Facing Fear	Substance Use
Preventing Burnout	Anxiety and Posttraumatic Stress Disorder
Realistic Optimism	Psychological First Aid
Self-Care	
Managing Moral Distress	
Finding Meaning and Purpose, Faith and Spirituality	
Building Social Connection, and Resilient Role Models	

**Table 2:** Demographic information.

	Total N = 1640	
Age 1548 (93%)	19 and Under	1 (<1%)
	20–29	636 (41%)
	30–39	508 (33%)
	40–49	207 (13%)
	50–59	111 (7%)
	60 or above	44 (3%)
	Prefer not to answer	19 (1%)
Gender 1554 (93%)	Female	972 (78%)
	Male	540 (35%)
	Other	14 (<1%)
	Prefer not to answer	30 (2%)
Race 1538 (92%)	White	460 (30%)
	Asian	399 (26%)
	Black	322 (21%)
	Latinx	253 (16%)
	Middle Eastern/North African	29 (2%)
	Native American/Alaskan Native	3 (<1%)
	Native Hawaiian/Other Pacific Islander	16 (1%)
	Other or Multiracial	63 (4%)
	Prefer Not to Answer	73 (5%)
Occupation 1640 (98%)	Nurse	651 (40%)
	Physician	298 (18%)
	Administrative	95 (7%)
	NP/PA	44 (4%)
	Security	175 (10%)
	Student	220 (13%)
	Other	157 (10%)
Work Setting 1619 (97%)	ED	487 (30%)
	ICU	253 (16%)
	Medical/Step Down	777 (48%)
	OB/Labor and Delivery	102 (6%)

Note: n’s are unweighted; percentages are weighted; NP = nurse practitioner; PA = physician assistant; ED = emergency department; ICU = intensive care unit; OB = Obstetrics.

**Table 3:** Post-huddle ratings.

Total Overall n = 1403					
n (%)					
Item	Strongly Disagree	Disagree	Neutral	Agree	Strongly Agree
1. The talk/workshop gave me tools to do my job well.	63 (4%)	23 (2%)	191 (14%)	627 (45%)	499 (36%)
2. The talk/workshop gave me tools that are relevant to my life outside of work.	62 (4%)	22 (2%)	187 (13%)	648 (46%)	484 (34%)
3. I feel more confident in my ability to “bounce back” from life’s challenges after the talk/workshop.	62 (4%)	45 (3%)	298 (21%)	600 (43%)	398 (28%)
4. I feel that the talk/workshops are one way that the hospital leadership shows they value me.	81 (6%)	81 (6%)	235 (17%)	595 (42%)	411 (30%)
5. I feel less stressed after completing the talk/workshops.	78 (6%)	84 (6%)	332 (23%)	545 (39%)	364 (25%)

## Data Availability

De-identified data for this project may be made available by reasonable request under a data-sharing agreement.
